# A Novel Anti-Inflammatory Role of Omega-3 PUFAs in Prevention and Treatment of Atherosclerosis and Vascular Cognitive Impairment and Dementia

**DOI:** 10.3390/nu11102279

**Published:** 2019-09-23

**Authors:** Marialaura Simonetto, Marco Infante, Ralph L. Sacco, Tatjana Rundek, David Della-Morte

**Affiliations:** 1 Department of Neurology and The Evelyn F. McKnight Brain Institute, University of Miami Miller School of Medicine, Miami, FL 33136, USA; marialaura.simonetto@yahoo.com (M.S.); rsacco@med.miami.edu (R.L.S.); trundek@med.miami.edu (T.R.); 2 Diabetes Research Institute (DRI) and Clinical Cell Transplant Program, University of Miami Miller School of Medicine, Miami, FL 33136, USA; marcoinfante.md@gmail.com; 3 Department of Systems Medicine, University of Rome “Tor Vergata”, 00133 Rome, Italy; 4 San Raffaele Roma Open University, 00166 Rome, Italy

**Keywords:** omega-3 PUFAs, omega-6 PUFAs, inflammation, resolvins, AA/EPA ratio, atherosclerosis, cardiovascular risk, neurodegeneration, vascular cognitive impairment and dementia

## Abstract

Atherosclerosis is an inflammatory chronic disease affecting arterial vessels and leading to vascular diseases, such as stroke and myocardial infarction. The relationship between atherosclerosis and risk of neurodegeneration has been established, in particular with vascular cognitive impairment and dementia (VCID). Systemic atherosclerosis increases the risk of VCID by inducing cerebral infarction, or through systemic or local inflammatory factors that underlie both atherosclerosis and cognition. Omega-3 and omega-6 polyunsaturated fatty acids (PUFAs) are involved in inflammatory processes, but with opposite roles. Specifically, omega-3 PUFAs exert anti-inflammatory properties by competing with omega-6 PUFAs and displacing arachidonic acid in membrane phospholipids, decreasing the production of pro-inflammatory eicosanoids. Experimental studies and some clinical trials have demonstrated that omega-3 PUFA supplementation may reduce the risk of different phenotypes of atherosclerosis and cardiovascular disease. This review describes the link between atherosclerosis, VCID and inflammation, as well as how omega-3 PUFA supplementation may be useful to prevent and treat inflammatory-related diseases.

## 1. Introduction to Omega-3 and Omega-6 PUFAs: An Overview of Their Metabolic Pathways

Polyunsaturated fatty acids (PUFAs) are fatty acids with two or more double bonds in their carbon chain. PUFAs can be further categorized according to the location of the first double bond relative to the terminal methyl group: omega-3 and omega-6 PUFAs are characterized by the presence of a double bond three and six atoms away from the methyl terminus, respectively. Omega-3 and omega-6 PUFAs represent the most biologically significant PUFAs and their role in cardiovascular, inflammatory, and metabolic diseases has been extensively studied [1,2,3,4].

**Figure 1:**
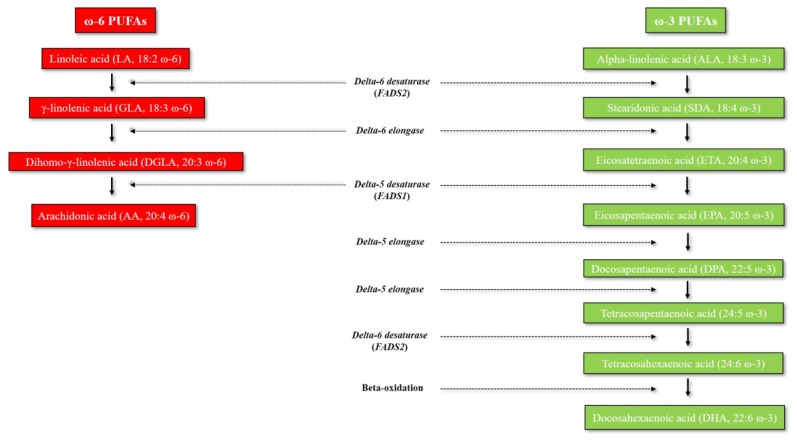
Metabolism of omega-3 and omega-6 PUFAs. Abbreviations: FADS, Fatty acid desaturase; PUFAs, polyunsaturated fatty acids.

Figure 1 illustrates the omega-6 and omega-3 PUFA biosynthetic pathway, which has already been extensively reviewed elsewhere [4,5]. Long-chain omega-3 and omega-6 PUFAs derive from alpha-linolenic acid (ALA, 18:3 ω-3) and linoleic acid (LA, 18:2 ω-6), respectively. ALA and LA are called essential fatty acids (EFAs), since mammals cannot synthetize them due to the lack of delta-12 and delta-15 desaturases, which are enzymes only present in marine algae and plants [6]. Humans must therefore obtain EFAs through dietary sources. The main dietary sources of ALA are represented by dairy products, and various seeds and seed oils (especially flaxseeds and walnut oil), whereas corn, safflower, and sunflower oils are particularly rich in LA. Humans are able to metabolize ALA and synthesize different downstream very long-chain and more unsaturated omega-3 PUFAs - including eicosapentaenoic acid (EPA, 20:5 ω-3), docosapentaenoic acid (DPA, 22:5 ω-3) and docosahexaenoic acid (DHA, 22:6 ω-3) - through multiple enzymatic elongation and desaturation reactions, which mainly occur in the liver (Figure 1) [5,7]. However, very long-chain omega-3 PUFAs can be also directly obtained through different dietary sources (cod liver oil, oily fish, algal oils) [5]. The omega-6 PUFA arachidonic acid (AA, 20:4 ω-6) can either be obtained from some dietary sources (especially meat, egg yolk and dairy products) or be synthesized endogenously from the omega-6 precursor LA through a series of enzymatic elongation and desaturation reactions (Figure 1) [4,8]. Delta-5 and delta-6 desaturases (encoded by *FADS1* and *FADS2* genes, respectively) are the rate-limiting enzymes for PUFA metabolism, and thus, the main determinants of PUFA levels [9].

## 2. Role of Omega-3 and Omega-6 PUFAs in Systemic Inflammation

**Figure 2:**
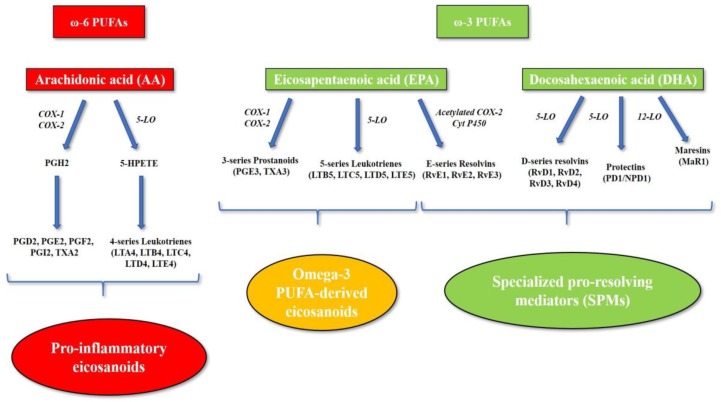
Eicosanoid and specialized pro-resolving mediator biosynthesis. The omega-6 PUFA arachidonic acid is the precursor of the pro-inflammatory eicosanoids. Cyclooxygenase and lipoxygenase enzymes catalyze the conversion of arachidonic acid into a series of pro-inflammatory mediators, including prostaglandins, thromboxanes and pro-inflammatory leukotrienes (4-series leukotrienes). The omega-3 PUFA eicosapentaenoic acid is also a substrate for arachidonic acid-cascade enzymes (cyclooxygenase and 5-lipoxygenase), leading to the production of alternative omega-3 PUFA-derived eicosanoids, such as 3-series prostanoids and 5-series leukotrienes, which are inactive metabolites or display lower pro-inflammatory activity compared to arachidonic acid-derived eicosanoids. Moreover, omega-3 PUFAs represent the precursors of a series of lipid mediators, including resolvins, protectins and maresins, which are collectively termed “specialized pro-resolving mediators” (SPMs). Abbreviations: 5-HPETE, 5-hydroperoxyeicosatetraenoic acid; 5-LO, 5-lipoxygenase; AA, arachidonic acid; COX-1, cyclooxygenase-1; COX-2, cyclooxygenase-2; Cyt P450, cytochrome P450; EPA, eicosapentaenoic acid; LO, lipoxygenase; LTA4, leukotriene A4; LTB4, leukotriene B4; LTB5, leukotriene B5; LTC4, leukotriene C4; LTC5, leukotriene C5; LTD4, leukotriene D4; LTD5, leukotriene D5; LTE4, leukotriene E4; LTE5, leukotriene E5; MaR1, maresin 1; NPD1, neuroprotectin D1; PD1, protectin D1; PGD2, prostaglandin D2; PGE2, prostaglandin E2; PGE3, prostaglandin E3; PGF2, prostaglandin F2; PGH2, prostaglandin H2; PGI2, prostacyclin; PUFA, polyunsaturated fatty acid; RvD1, resolvin D1; RvD2, resolvin D2; RvD3, resolvin D3; RvD4; resolvin D4; RvE1, resolvin E1; RvE2, resolvin E2; RvE3, resolvin E3; SPMs, specialized pro-resolving mediators; TXA2, thromboxane A2; TXA3, thromboxane A3.

Lipid mediators derived from the omega-6 PUFA AA are involved in inflammation at different stages. In particular, the initiation of acute inflammation is regulated by several lipid mediators, including the eicosanoids prostaglandins (PGs), thromboxanes (TXs) and leukotrienes (LTs), which play a pivotal role in the modulation of blood flood, endothelial permeability, polymorphonuclear neutrophil (PMN) chemotaxis, and platelet aggregation [10,11]. Cyclooxygenase (COX) and lipoxygenase (LO) enzymes catalyze the conversion of AA into a series of pro-inflammatory mediators, including PGs, prostacyclin (PGI2), TXA2 and pro-inflammatory leukotrienes, which are also known as 4-series leukotrienes (Figure 2) [12,13].

On the other hand, omega-3 PUFAs exert anti-inflammatory properties by competing with omega-6 PUFAs and displacing AA in membrane phospholipids, reducing the production of pro-inflammatory eicosanoids. In particular, EPA is also a substrate for AA cascade enzymes (COX, 5-LO), leading to the production of alternative omega-3 PUFA-derived eicosanoids, such as 3-series prostanoids and 5-series leukotrienes. Despite being similar in structure and stability, EPA-derived eicosanoids are inactive metabolites and display lower pro-inflammatory activity compared to AA-derived eicosanoids [14,15,16,17]. However, omega-3 PUFAs play an anti-inflammatory role especially by promoting the resolution of inflammation. In fact, they are precursors of a series of lipid mediators, including resolvins, protectins and maresins, which are collectively termed “specialized pro-resolving mediators” (SPMs) [18]. SPMs are produced by PMN and macrophages during the resolution of inflammation, stimulating key cellular events, such as the cessation of PMN infiltration, macrophage switching to anti-inflammatory phenotype M2, and apoptotic cell clearance [18,19]. SPMs are synthesized through a complex series of enzymatic reactions mediated by acetylated COX-2, P450, and LO enzymes. EPA represents the precursor of the E-series resolvins (RvE1, RvE2, and RvE3), whereas DHA leads to the production of three distinct families of SPMs, namely D-series resolvins (RvD1, RvD2, RvD3, RvD4), protectins (protectin D1, known as neuroprotectin D1 [NPD1] when formed in the nervous system) [20], and maresins (MaR1) (Figure 2) [18,20].

As mentioned above, the majority of omega-3 and omega-6 PUFAs are ingested through the diet. Due to the dramatic changes observed in dietary pattern over decades, Western diets have progressively evolved towards an increased amount of omega-6 PUFAs compared to omega-3 PUFAs. The dietary imbalance between omega-6 and omega-3 PUFAs has therefore been suggested to play a role in triggering systemic inflammation, and thus increasing the risk for several chronic diseases, such as obesity, cardiovascular disease (CVD) and autoimmune disorders [21,22,23]. In particular, omega-6 to omega-3 serum ratio in adults has gradually increased to values ranging from 15:1 to 25:1, which are markedly high compared to those prevalent in the diet of our ancestors (omega-6 to omega-3 ratio of around 1:1) [22,23,24,25]. The omega-6 PUFA LA represents the main PUFA in most Western diets and is consumed in 5- to 20-fold higher amount than the omega-3 PUFA ALA [26]. Importantly, omega-3 and omega-6 PUFAs compete for the same desaturation enzymes [23,27,28,29]. Even if both delta-5 and delta-6 desaturases prefer ALA to LA as a substrate, a high dietary LA intake – such as that observed in most Western diets – interferes with the desaturation and elongation of ALA to long-chain omega-3 PUFAs (EPA, DPA, and DHA) [28,29,30]. 

## 3. Pathogenesis of Atherosclerosis and Vascular Cognitive Impairment and Dementia: Role of Inflammation

The National Institute of Health has defined vascular contributions to cognitive impairment and dementia (VCID) as conditions arising from stroke and other vascular brain injuries that cause significant changes to memory, thinking and behavior [31]. VCID encompasses a series of alterations in cognitive function (from the deterioration of decisional and executive abilities to impairment in multiple cognitive domains that affect activities of daily living) that are caused by vascular risk factors. The term VCID includes two forms of the same condition: vascular dementia (VD) and vascular cognitive impairment (VCI), both arising as a result of risk factors for cerebrovascular disease (e.g., atrial fibrillation, diabetes, hypertension, and dyslipidemia) [32].

The central role of inflammation in atherosclerosis, and thus in cardiovascular (CV) risk and vascular cognitive impairment and dementia, has been well established [33,34,35,36]. Atherosclerosis is a chronic inflammatory syndrome that leads to the progressive thickening of large artery walls [37] and reduced blood flow over time, which can result in stroke, myocardial infarction (MI) and VCID [38]. In the past, atherosclerotic symptoms were thought to be exclusively dependent on the degree of stenosis determining the impaired perfusion of target tissues, such as brain and heart [6]. However, recent clinical evidence showed that thrombotic complications, that often lead to stroke and MI, may not exclusively result from critical stenosis. This new knowledge prompted a shift in our understanding of the atherosclerotic disease, and led to the recognition of the central role of inflammation in the atherosclerotic process [39]. The inflammatory process that leads to atheromatous plaque has been extensively described, and even if the initiation of atherosclerosis is still not fully elucidated, it has been showed that leukocyte recruitment and production of pro-inflammatory cytokines represent the main triggers during the early phase of atherogenesis [34,40]. Atherosclerosis begins with inflammatory changes within the endothelium caused by low-density lipoproteins (LDLs) being deposited on endothelial cells [41,42]. The activated endothelial cells produce chemokines and chemoattractant proteins to recruit monocytes and leukocytes to the intima of the vessels. The recruited monocytes differentiate into macrophages that, in turn, internalize LDLs particles and generate foam cells. It is becoming increasingly clear that the deposition of LDLs into the arterial walls is not sufficient to trigger atherogenesis, rather it is the subsequent inflammatory response, promoted by monocytes and leukocytes, that initiates the atherosclerotic process [43]. The crucial step in atherogenesis has been identified when invading monocytes differentiate into macrophages in the intima of the vessels, and while internalizing and accumulating intracellularly oxidized-LDLs, they gradually transform into foam cells [44]. Over time, clusters of foam cells become fatty-streaks in the intima of the endothelium [45]. As the disease progresses, some of the endothelial cells adjacent to the atheromatous endothelium begin to harden, forming a solid core called the fibrous cap of the atheroma, which begins to enlarge and partially occlude the vessel to cause a progressive narrowing of the arterial lumen that leads to stroke and VCID [46]. Atherosclerosis complications may occur when the foam cells inside the atheromatous plaque secrete pro-inflammatory cytokines, reactive oxygen species (ROS) and other mediators that can cause death of macrophages, forming the necrotic core of the mature plaque. Macrophages and phagocytes produce matrix metalloproteinases (MMPs) that degrade the plaque’s fibrous cap, permitting the blood to contact the plaque’s necrotic core and leading to the thrombotic complications, such as stroke and MI [34,40]. Vascular smooth muscle cells (VSMCs) also play a role in the pathogenesis of atherosclerosis. Notably, VSMCs undergo a phenotypic switching resulting in increased capacity for cell proliferation and migration. In addition, VSMCs can also switch to macrophage-like cells, which display a reduced phagocytic capacity and directly promote the formation of the necrotic core of the plaque [47].

It has been shown that the progressive build-up of atherosclerotic plaque in the cerebral arteries can contribute to the development of VCID either directly or as a consequence of stroke. Since it has been proven that atherosclerosis is triggered by inflammation, it might be important to target inflammation to reduce the burden of VCID in older adults.

In the vascular inflammatory process, inflammatory cytokines generate proteases, endothelial adhesion molecules, and other mediators that ultimately induce the production of interleukin (IL)-6, which stimulates the production of acute-phase reactants such as C-reactive protein by the liver [48], and triggers a systemic inflammatory reaction. In addition, platelets and perivascular adipose tissue can generate inflammatory mediators, that also play an important role in the atherothrombotic process [48]. The discovery of the pivotal role of inflammation in atherosclerosis has prompted the recognition of inflammatory biomarkers of CV risk (e.g., C-reactive protein, soluble CD40 ligand, adiponectin, IL-18, and MMP-9) and has led to the adoption of these biomarkers for CV risk prediction [49]. In the randomized, double-blind, placebo-controlled trial Canakinumab Anti-inflammatory Thrombosis Outcome Study (CANTOS), which involved stable patients with previous MI, a significant reduction in major CV events compared to placebo was achieved by anti-inflammatory therapy targeting IL-1β with the monoclonal antibody canakinumab [50]. Other studies that aim to target inflammatory biomarkers for primary or secondary prevention of CV events are currently ongoing. The Cardiovascular Inflammation Reduction Trial (CIRT) investigated the role of inflammation on atherothrombosis in patients with prior MI, type 2 diabetes or metabolic syndrome and found that methotrexate was not effective in reducing atherosclerosis [51]. The Colchicine Cardiovascular Outcomes Trial (COLCOT; ClinicalTrials.gov Identifier: NCT02551094) is evaluating whether the long-term treatment with colchicine is able to reduce the rate of CV events in patients after MI. The extensive evidence of the crucial role of inflammation in the development of atherosclerosis and the recent evidence of the role of omega-3 and omega-6 PUFAs in the inflammatory process may open a relevant alternative for the primary and secondary prevention of CV diseases and VCID.

## 4. The Role of Omega-3 and Omega-6 PUFAs in Atherosclerosis, Cardiovascular Disease and Vascular Inflammation

**Figure 3:**
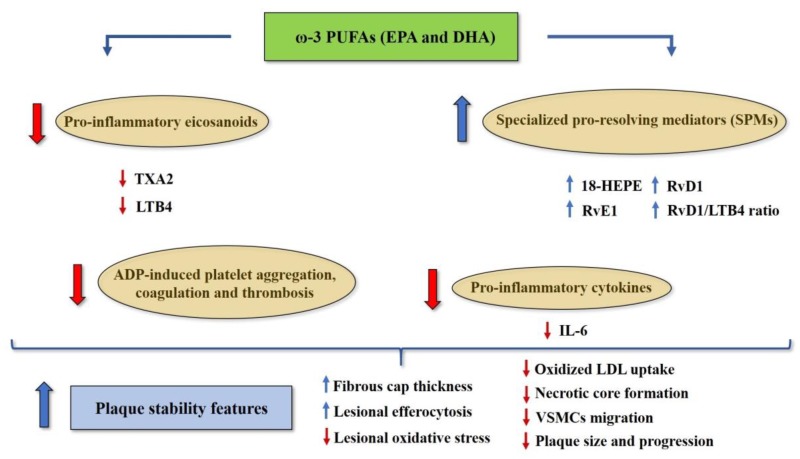
Potential mechanisms underlying the protective effects of omega-3 PUFAs EPA and DHA against vascular inflammation and atherosclerosis. Abbreviations: 18-HEPE, 18-monohydroxy EPA; ADP, adenosine diphosphate; EPA, eicosapentaenoic acid; DHA, docosahexaenoic acid; IL-6, interleukin-6; LDL, low-density lipoprotein; LTB4, leukotriene B4; RvE1, resolvin E1; RvD1, resolvin D1; SPMs, specialized pro-resolving mediators; TXA2, thromboxane A2; VSMCs, vascular smooth muscle cells.

Experimental evidence strongly suggests the involvement of omega-3 PUFA and their metabolites SPMs in the resolution of inflammation in atherosclerosis. Notably, omega-3 PUFAs may reduce the inflammatory pathway in atherosclerosis by both reducing production of pro-inflammatory eicosanoids, as well as by increasing synthesis of SPMs (Figure 3) [52]. Interestingly, Fredman et al. showed that vulnerable regions of human carotid atherosclerotic plaques exhibit significantly lower levels of pro-resolving mediator RvD1, as well as significantly higher levels of pro-inflammatory mediator LTB4 [53]. The authors also demonstrated that the exogenous administration of RvD1 to fat-fed LDLR^−/−^ mice during atherosclerotic plaque progression was able to restore the RvD1/LTB4 ratio to that of less advanced atherosclerotic lesions, and to promote plaque stability features (e.g., thicker fibrous caps, improved lesional efferocytosis, and reduced lesional oxidative stress and necrosis) [53]. Accordingly, Thul et al. found that a higher salivary RvD1/LTB4 ratio was significantly associated with lower intima media thickness in individuals with subclinical atherosclerosis [54]. 

Moreover, a recent experimental study has suggested that EPA supplementation may reduce diet-induced atherosclerosis in ApoE^−/−^ mice [55]. This study also showed a significant increase in omega-3 PUFA content in different tissues (myocardium, spleen, and skeletal muscle), which was accompanied by a parallel reduction in omega-6 PUFA content at the same level. Also, 18-monohydroxy EPA (18-HEPE, the direct precursor for RvE1 synthesis) was identified as the most prominent circulating EPA-derived metabolite after EPA supplementation, and deletion of the RvE1 receptor Erv1/Chemr23 was associated with pro-atherogenic signaling in macrophages, reduced phagocytosis, increased oxidized LDL uptake, increased atherosclerotic plaque size, and necrotic core formation, in the absence of changing on total cholesterol and triglyceride serum levels [55]. Overall, these findings suggest that EPA may exert protective effects in atherosclerosis through its derived SPM RvE1, regardless of its effects on total cholesterol and triglyceride levels. Intriguingly, RvE1 has also been shown to attenuate injury-induced vascular neointimal formation in mice by inhibition of inflammatory responses and VSMCs migration [56].

In addition, Yamano et al. showed that patients with coronary atherosclerotic plaque supplemented with EPA (at a dose of 1.8 g/day) for eight months exhibited a significant increase in the fibrous cap thickness (assessed by optical coherence tomography), which was accompanied by a significant increase in serum EPA/AA ratio compared to the control group [57]. Other studies showed that lower EPA/AA ratio and omega-3/omega-6 PUFAs ratio represent independently associated factors of carotid atherosclerosis and high-risk atherosclerotic coronary plaques [58,59].

Cross-sectional studies showed that the omega-3 index (defined as the omega-3 PUFA content of red blood cells) is inversely associated with inflammatory biomarkers (C-reactive protein and IL-6) in patients with peripheral artery disease and stable coronary artery disease (CAD) [60,61]. Moreover, Massaro et al. found that DHA attenuates the in vitro endothelial expression of COX-2 [62], which is increased in human atherosclerotic lesions [63].

Wang et al. conducted a brilliant study on LDL-receptor null mice fed different high saturated fat diets differing only in the omega-6/EPA+DHA ratio. The authors found that mice fed with the lowest omega-6/EPA+DHA ratio diet (ratio = 1:1) exhibited significantly lower circulating levels of non-HDL cholesterol and IL-6 compared to mice fed with the diet with the highest omega-6/EPA+DHA ratio [64].

Evidence also suggests that omega-3 PUFAs may reduce platelet aggregation, coagulation and thrombosis, without increasing the risk of bleeding [30]. A meta-analysis of 15 Randomized Controlled Trials (RCTs) demonstrated that omega-3 PUFA supplementation is associated with a significant reduction in adenosine diphosphate (ADP)-induced platelet aggregation [65]. Interestingly, a cross-over study conducted on healthy subjects showed that platelet aggregation in vitro decreases as the dietary LA/ALA ratio also decreases [66]. These findings may rely on the fact that omega-3 PUFAs EPA and DHA can be incorporated into platelet phospholipids membrane at the expense of AA, thus decreasing the synthesis of AA-derived metabolite TXA2 and reducing platelet aggregation [67]. Figure 3 illustrates the potential mechanisms underlying the protective effects of omega-3 PUFAs (EPA and DHA) against vascular inflammation and atherosclerosis.

Despite the afore mentioned experimental evidence, results from clinical studies on the role of omega-6 and omega-3 PUFAs in vascular inflammation, atherosclerosis and CVD are still controversial. In fact, a meta-analysis of observational and intervention studies reporting information on biomarkers of omega-3 and omega-6 PUFAs found that circulating levels of EPA, DHA and also AA were significantly associated with a lower risk for coronary events [68]. With regard to fatty acid intake, dietary intake of omega-6 PUFAs — which predominantly consisted of LA — was not significantly associated with risk for coronary events [68].

Furthermore, several studies investigated the clinical utility of omega-6 to omega-3 ratio in CV risk, and whether the reduction in this ratio may have an impact on CV risk by decreasing the competitive influence of LA on ALA metabolism to its longer chain down-stream products, including EPA, DPA, and DHA [30]. Nevertheless, in 2006 the UK Food Standards Agency (FSA) Workshop documented that omega-6 to omega-3 ratio is of limited usefulness in the context of CV health and CVD due to a number of reasons [69]. First, the ratio cannot distinguish between specific omega-3 (e.g., ALA, EPA, and DHA) or omega-6 PUFAs (e.g., LA and AA), which are not physiologically equivalent in terms of metabolic and pro- or anti-inflammatory activity. Second, a given ratio value may be the result of different directional changes in omega-3 and/or omega-6 PUFA levels. For instance, the same magnitude of increase in omega-6 to omega-3 ratio can derive from an increase in omega-6 intake (with no change in omega-3 intake), or from a reduction in omega-3 intake (with no change in omega-6 intake). In this context, the ratio may assume that higher levels of omega-6 PUFAs and lower levels of omega-3 PUFAs have the same impact CV risk. Nonetheless, higher tissue levels of omega-6 (e.g., LA) and omega-3 PUFAs (e.g., EPA and DHA) have both been associated with reduced CV risk [70], whereas lower tissue levels of omega-3 PUFAs (particularly EPA and DHA) have been associated with increased CV risk [70,71]. Finally, the Quantification of the Optimal n-6/n-3 Ratio in the UK Diet (OPTILIP) study [72,73] and a stable isotope tracer study [74] independently concluded that omega-6 to omega-3 ratio is of no value in modifying CV risk [75].

Therefore, additional biomarkers have been suggested as more useful tools to assess the fatty acid intake and to relate the fatty acid intake to clinical outcomes. In 2004, Harris and Von Schacky proposed the omega-3 index as a novel risk factor for death from coronary heart disease [76]. Omega-3 index is defined as the content of EPA and DHA in red blood cell membranes, expressed as a percentage of total fatty acids. In particular, omega-3 index scores of ≥8% or ≤4% were associated with the highest and lowest cardioprotection, respectively [76]. Thus, it has been suggested that clinical trials evaluating the role of omega-3 PUFA intake or supplementation in CV health should include the omega-3 index in the study design, recruit individuals with a low index, and treat them within a specific target range (e.g., ≥8%) in order to give rise to more accurate findings [77]. Harris et al. examined the relationship between erythrocyte omega-3 PUFA levels among 2500 older participants in the Framingham Heart Study’s Offspring cohort. Interestingly, the authors found that participants in the highest (>6.8%) omega-3 index quintile exhibited a 35% lower risk for total mortality, as well as a 39% lower risk for incident CVD compared to those in the lowest omega-3 index quintile (<4.2%) [78]. 

## 5. The Role of Linoleic Acid (LA) in Atherosclerosis and Cardiovascular Disease: Evidence and Controversies

LA is the main omega-6 PUFA found in vegetable oils, whose consumption has dramatically increased over the last decades in the Western world. Indeed, a systematic literature review of studies measuring the concentration of LA in subcutaneous adipose tissue—which accurately reflects dietary LA intake—revealed a dramatic increase in adipose tissue LA over the last half century in the USA [79]. Importantly, the amount of LA in adipose tissue and in platelets has been positively associated with the degree of CAD [80]. Animal studies showed that LA can increase the expression of vascular cell adhesion molecule-1 (VCAM-1) and intercellular cell adhesion molecule-1 (ICAM-1) through the nuclear factor (NF)-κB pathway in the aorta [81]. Moreover, LA has also been shown to increase LDL transfer across cultured endothelial monolayers, which is regarded as a crucial step in the atherosclerosis process [82]. A cross-sectional study conducted by Schwertner and Mosser found higher LA concentrations in phospholipid fatty acids and serum cholesteryl esters in patients with CAD compared to those without CAD (sample size: *n* = 30 men; 18 subjects with CAD and 12 subjects without CAD) [83]. Thereafter, another cross-sectional study conducted by Jira et al. showed that concentrations of oxidized LA metabolites are significantly higher in LDL of atherosclerotic subjects compared to those of healthy individuals (sample size: *n* = 36 subjects; 17 atherosclerotic patients and 19 healthy volunteers) [84]. Reaven et al. showed that linoleate-enriched diet increased the susceptibility to oxidation of LDL and HDL in hypercholesterolemic subjects [85]. Altogether, these findings suggested that the oxidation of LDL—which is considered the crucial factor for the development of early atherosclerotic lesions [86]—may be initiated by the oxidation of LA contained within LDL particles [87], thus generating the “oxidized LA hypothesis” of atherosclerosis and CVD [30,88]. 

Another concern that has been raised about excessive LA intake regards the potential conversion of LA into AA, resulting in a higher production of pro-inflammatory eicosanoids [89], which may be associated with atherosclerosis and increased CV risk [30,90]. Therefore, lowering dietary LA intake has been suggested as a useful strategy to reduce tissue AA levels, decrease eicosanoid synthesis and prevent the production of oxidized LA metabolites, as it has also been demonstrated in clinical studies [30,88,91]. Nevertheless, the conversion of LA into AA appears to be strictly regulated, since even high dietary intakes of LA do not substantially modify tissue content of AA [92,93]. In addition, the influence of dietary intake of LA and ALA on omega-3 PUFA metabolism is still under debate. In this context, the hypothesis that a high omega-6 to omega-3 ratio in the diet decreases the conversion of ALA into its derivatives EPA and DHA has been tested in human subjects. Goyens et al. conducted a study on 29 healthy volunteers, who received a control diet with a LA to ALA ratio of 19:1 for 4 weeks [74]. Thereafter, the participants were randomized to receive for six weeks the same control diet (control group) or two diets with the same LA to ALA ratio (7:1), designed by either decreasing LA and keeping ALA constant (low-LA diet) or by increasing ALA and keeping LA constant (high-ALA diet). Interestingly, the conversion rate of ALA into EPA and DHA was assessed at the end of each period using an ALA stable isotope tracer and measuring omega-3 PUFA concentrations in fasting plasma phospholipids. If the aforementioned hypothesis were true, the metabolic parameters of the low-LA and the high-ALA diets should have been similar, since both diets had the same LA to ALA ratio (7:1). Nonetheless, the conversion rate of ALA into EPA increased significantly in the low-LA diet, whereas it decreased in the high-ALA diet group. On the contrary, the synthesis of DHA increased significantly in the high-ALA diet group, where it did not change significantly in the low-LA diet group compared to the control group. These findings support that the absolute amounts of LA and ALA in the diet, rather than the LA to ALA ratio (a surrogate marker of omega-6 to omega-3 ratio), determine the conversion of ALA into EPA and DHA [74]. However, increased dietary intake of ALA at the expense of LA has been shown to have small benefit in altering EPA and DHA status or improving CV risk and inflammatory markers [26]. 

On the other hand, the pro-inflammatory properties of LA and its role in modulating CV risk still remain highly controversial. A meta-analysis of RCTs showed that omega-6 specific PUFA diets (including safflower and/or corn oils, which contain high amounts of LA and small proportions of ALA) significantly increased the risk of non-fatal MI plus death related to coronary heart disease in comparison with mixed omega-3/omega-6 diets [94]. Conversely, some evidence suggests that LA may have a cardioprotective role. In fact, observational studies and RCTs documented an inverse association between omega-6 PUFA intake (primarily LA intake) and risk of coronary heart disease [95,96,97] and CV mortality [98]. Moreover, case-control studies showed that lower tissue/serum LA levels were inversely associated with the risk of stroke [99] and non-fatal CV events [70]. A meta-analysis of prospective studies reported that dietary intake of LA was significantly associated with lower risk of coronary events and deaths related to coronary heart disease in a dose-response manner [100]. Another meta-analysis showed that tissue and blood LA concentrations were inversely associated with risk for coronary events [70]. Additionally, a recent pooled analysis of 30 prospective observational studies reported that higher circulating and tissue levels of LA were associated with a significant lower risk of total CVD, cardiovascular mortality, and ischemic stroke, without heterogeneity in population subgroups across studies [101]. In keeping with these findings, a systematic review of RCTs showed that dietary intake of LA did not lead to an increase in inflammatory markers, including C-reactive protein, tumor necrosis factor-α, fibrinogen, plasminogen activator inhibitor type 1, and soluble vascular adhesion molecules [102]. 

In conclusion, evidence from human studies shows that LA is not harmful to CV health, but it may play a beneficial role in prevention of CVD. However, mechanistic studies are needed in order to understand the exact mechanisms underlying the cardioprotective effects of LA. 

## 6. Omega-3 PUFA Supplementation for Primary and Secondary Prevention of Atherosclerosis and Cardiovascular Disease: Lessons from Clinical Trials

Different large-scale randomized placebo-controlled trials investigated the effects of omega-3 PUFA supplementation in terms of primary and secondary prevention of CVD. The Japan EPA Lipid Intervention Study (JELIS) study first aimed to assess whether the long-term supplementation with EPA was effective for prevention of major coronary events [103]. The study enrolled 18,645 Japanese hypercholesterolemic patients, who were randomized to receive either statin alone or EPA (at a dose of 1800 mg/day) in addition to statin during a five-year follow-up period. Patients in the EPA group showed a 19% relative reduction in major coronary events compared to those on statin alone (*p* = 0.011). Subgroup analyses revealed that EPA supplementation led to a reduction in major coronary events in both patients with and without history of CAD, although the statistical significance was reached only in the secondary prevention subgroup (Table 1). Interestingly, the AA/EPA ratio (1.6 in both groups at baseline) decreased from 1.6 at baseline to 0.8 at the end of the study in the EPA group, whereas it remained unchanged in the control group [103]. Subsequent post-hoc analyses of the JELIS study demonstrated that higher plasma levels of EPA are inversely associated with the risk of major coronary events [104,105], especially in individuals with prior MI [105]. Matsuzaki et al. also found that the incidence of cardiac death or MI in the JELIS cohort was significantly lower among patients with the highest EPA/AA ratio compared to those with the lowest ratio (adjusted HR 0.58, *p* = 0.038) [105]. However, these findings should be interpreted cautiously, since they are based on a post-hoc analysis that has evaluated the data from JELIS as an observational study.

In 2010, Kromhout et al. published the results of the Alpha Omega Trial, a multicenter, placebo-controlled trial examining the effect of the daily intake of margarine supplemented with different types of omega-3 PUFAs on the rate of CV events among patients with history of MI [106]. The authors found that neither margarine enriched with EPA and DHA, nor margarine enriched with ALA were able to significantly reduce the rate of major CV events [106] (Table 1). Similar findings were observed in the Supplémentation en Folates et Omega-3 (SU.FOL.OM3) trial, which showed that daily low-dose omega-3 PUFA supplementation had no significant effects on major CV events in patients with a history of unstable angina, MI, or ischemic stroke [107] (Table 1). Moreover, the outcome reduction with an initial glargine intervention (ORIGIN) trial sought to determine the effect of omega-3 PUFA supplementation in patients at increased CV risk (defined as history of MI, angina with documented ischemia, stroke, or revascularization) who had diabetes, impaired glucose tolerance or impaired fasting glucose [108]. The study enrolled 12,536 participants who were randomly assigned to receive a 1-g daily capsule containing either EPA and DHA or placebo. The primary outcome was death from CV causes. However, no significant between-group differences were observed in the primary outcome during a median follow-up of 6.2 years. Moreover, omega-3 PUFA supplementation did not show a significant effect on the rate of major CV events, death from arrhythmia, or death from any cause [108] (Table 1). Thereafter, the Risk and Prevention Study found that EPA and DHA supplementation at a dose of 1 g/day did not significantly reduce CV mortality and morbidity among patients with multiple CV risk factors or clinical evidence of atherosclerotic vascular disease without history of previous MI [109] (Table 1). 

With regard to the primary prevention of CVD, the multicenter, randomized, placebo-controlled trial ASCEND (A Study of Cardiovascular Events in Diabetes) investigated whether omega-3 PUFA supplementation exerted an effect on CV events in patients with diabetes during a mean follow-up of 7.4 years [110]. Of note, 15,480 patients with diabetes and no evidence of CVD were randomly assigned to receive a 1-g daily capsule containing EPA and DHA or placebo. The primary outcome was a first serious vascular event (defined as a composite of non-fatal MI or stroke, transient ischemic attack, or vascular death, excluding confirmed intracranial hemorrhage), whereas the secondary outcome was a composite of any serious vascular event or any revascularization procedure. At the end of the study, no significant between-group differences were observed in primary and secondary outcomes (Table 1), suggesting that omega-3 PUFA supplementation at the afore mentioned daily dose does not significantly modify the risk of CV events [110]. In addition, the Vitamin D and Omega-3 (VITAL) trial has recently evaluated the effects of omega-3 PUFA supplementation (at a dose of 1 g/day, administered as a fish-oil capsule containing both EPA and DHA) in primary prevention of CVD during a median follow-up of 5.3 years [111]. The study population included 25,871 participants with no history of CVD. The primary endpoint consisted of major CV events (a composite of MI, stroke, or death from CV causes). Omega-3 PUFA supplementation did not result in lower incidence of major CV events (defined as a composite endpoint of MI, stroke, or death from CV causes) compared to placebo. However, the analyses of secondary end points showed that omega-3 PUFA supplementation was associated with a significant 28% reduction in risk for total MI compared to placebo [111] (Table 1).

On the other side, the large, randomized, placebo-controlled trial REDUCE-IT (Reduction of Cardiovascular Events with Icosapent Ethyl–Intervention Trial) assessed the effects of high dose (4 g/day) icosapent ethyl—a highly purified EPA ethyl ester—on CV risk among hypertriglyceridemic patients with established CVD or diabetes and other risk factors, who had been receiving statin therapy [112]. The majority of patients (70.7%) were enrolled for secondary prevention of CV events. Importantly, the authors found that supplementation with icosapent ethyl led to a significant reduction in the rates of both primary and secondary endpoints compared to placebo [112] (Table 1). Interestingly, Braeckman et al. previously demonstrated that hypertriglyceridemic patients taking the same dose of icosapent ethyl (4 g/day) used in the REDUCE-IT trial exhibited a significant reduction in the AA/EPA ratio after 12 week-supplementation (from 12.3 at baseline to 1.2 at the end of the study; *p* < 0.0001) [113].

Altogether, these findings suggest that omega-3 PUFAs may have a beneficial role in terms of prevention of CVD when administered at higher doses. In fact, a possible explanation for the lack of CV benefits from omega-3 PUFA supplementation in most of the CV outcomes trials may be due to the relatively low doses administered (≤1 g/day) [114]. Indeed, JELIS and REDUCE-IT showed that high dose omega-3 administration (between 1.8 and 4 g/day) led to a significant reduction in CV morbidity and mortality. Therefore, it may be speculated that beneficial effects of omega-3 PUFAs at high doses could be mediated, at least in part, by their protective properties against inflammatory, thrombotic and atherosclerotic processes [115,116]. This may be due to the fact that high doses of omega-3 PUFAs could be needed in order to achieve high circulating levels of SPMs, as it has been previously demonstrated [117]. However, no studies have been conducted in order to address how markers of SPM synthesis (e.g., Rv/leukotriene ratio) and circulating levels of SPMs relate to the intracellular effects of these pro-resolving mediators.

Among the afore mentioned RCTs, only the REDUCE-IT trial investigated the effects of omega-3 PUFA supplementation on pro-inflammatory biomarkers, showing a significant reduction in circulating levels of hsCRP (high-sensitivity C-reactive protein) in the omega-3 group compared to placebo (Table 1). Therefore, the effects of (high dose)-omega-3 PUFAs and their derivative metabolites SPMs on inflammatory biomarkers and CV outcomes remain to be extensively investigated in future mechanistic studies and randomized trials.

## 7. Role of Omega-3 and Omega-6 PUFAs in Neuronal Cells

The fatty acid composition in the brain is unique: the neuronal cells are characterized by high levels of palmitate (a saturated fatty acid), omega-6 PUFA, but low levels of omega-3 PUFAs [118]. Among the omega-3 PUFAs found in the brain tissue, the quantitatively most important omega-3 PUFA is DHA, which is 250–300 times more abundant compared to EPA [119]. The cerebral synthesis of EPA and DHA is low, suggesting that the brain maintains fatty acid levels via uptake from dietary or liver sources [120]. In fact, EPA and DHA can cross the blood-brain barrier by diffusion [121]. The brain levels of EPA are maintained low by several mechanisms, including decreased incorporation, beta-oxidation and lower phospholipid recycling [122,123]. The high levels of DHA are quite conserved across species, suggesting a specific role of DHA in the neuronal membrane [118]. It has been shown that DHA carries a greater tendency to accumulate in sphingomyelin/cholesterol-rich lipid rafts compared to EPA [124], and thus has a greater potential to affect neuronal signaling [125].

DHA has been proven to have an indispensable role in neuronal membranes. The omega-3 PUFA dietary deficiency studies showed how the reduction of brain DHA can produce alterations in neuronal membrane properties [126] and in enzyme activity and electrophysiological properties [127], alter neurotransmission [128], and decrease spatial memory performance [129].

## 8. Role of Omega-3 and Omega-6 PUFAs in Alzheimer’s Disease and Vascular Cognitive Impairment and Dementia 

Extensive evidence shows that aging is characterized by alteration in energy metabolism, which includes increased oxidative stress and increased inflammation, that may lead to increased atherosclerosis susceptibility [130,131]. Structural changes, such as a reduction in total brain volume and altered neuronal membrane lipid content, have been described in aging populations [132]. For these reasons, the aging brain is more susceptible to develop atherosclerosis, which can ultimately lead to VCID and to development of neurodegenerative diseases, including Alzheimer’s disease (AD). PUFAs can modulate many signal transduction pathways in neuronal cells. Therefore, age-dependent neurodegeneration may be prevented by controlling these pathways. Lessons from animal models have shown that the ratio between omega-3 and omega-6 PUFAs influences various aspects of serotoninergic and catecholaminergic neurotransmission, that are the first to be lost during AD. When Phospholipase A2 (PLA2) hydrolyzes fatty acids from membrane phospholipids, it liberates omega-6 PUFAs, which are metabolized to prostaglandins with a higher inflammatory potential compared with those generated from omega-3 PUFAs. Thus, the activity of PLA2, coupled with the altered membrane fatty acid composition typical of aging, may play a central role in the development of neuronal dysfunction. In particular, PUFAs increase both phospholipase C (PLC) and protein kinase C (PKC) activities on neuronal membranes, which are involved in alpha-1 adrenergic transmission [133,134] and can modulate PLA2, that ultimately regulates the production of prostaglandins, thromboxane, and leukotrienes [135,136]. Intervention trials in human subjects have shown that omega-3 PUFAs have possible positive effects in the treatment of various psychiatric disorders. Several studies have investigated the association between omega-3 PUFA (EPA and DHA) levels and risk of dementia and cognitive decline. Samieri et al. showed that high levels of EPA, but not DHA, are associated with lower hippocampal, para-hippocampal and amygdala atrophy, along with slower cognitive decline and dementia risk in older adults [137,138,139,140]. In a recent meta-analysis, Lin et al. showed that omega-3 PUFA levels are significantly reduced in dementia patients, but only levels of EPA are significantly lower in pre-dementia patients [141]. Moreover, Schaefer et al. described a protective effect of high plasma levels of DHA (but not EPA) in dementia prevention (participants with higher DHA levels showed a 47% risk reduction of developing dementia) [142]. Tan et al., in a cross-sectional study conducted on 1575 dementia-free participants, described how lower levels of DHA (but not EPA) were associated with worse memory and executive function performance [143]. Salem et al. described the role of DHA in the nervous system and its regulatory role in the G-protein signaling. The authors showed that DHA has a protective role against apoptosis and plays a role in decreasing phosphatidylserine levels, that, conversely, control cell signaling and cell proliferation [144]. An anti-amyloidogenic role of DHA has been described by Dyall et al., that showed how DHA can increase neuronal membrane fluidity, decrease membrane peroxidation by reducing cholesterol levels in the cell membrane that leads to reduced oxidative stress in the cerebral cortex and in the hippocampus, and ultimately decrease the learning disabilities related to AD [145]. Moreover, a DHA transporter has been recently identified on the blood-brain barrier [146]. Both these newly discovered properties of DHA (its anti-amyloidogenic properties and its ability to cross the blood-brain barrier) are crucial in the pathogenesis and in the prevention of AD and VCID. Finally, recent evidence shows that omega-3 PUFA (EPA and DHA) deficiency may lead to reduced brain glucose uptake [147] and, in turn, higher memory impairment [148].

Briefly, PLA2 hydrolyzes fatty acids from membrane phospholipids, liberates dihomo-gamma-linolenic acid (DGLA) and AA (omega-6 PUFAs), which are then metabolized to prostaglandins and thromboxane, that have pro-inflammatory properties [149]. It has been shown that PLA2 activity and omega-6 PUFAs may play a role in neuronal dysfunction, and in particular a highly reactive PLA2 coupled with high omega-6 PUFAs content in the cell membrane has been associated with a higher inflammatory state and it has been found in various psychiatric disorders [150,151]. This explains the crucial role of a balanced ratio between omega-3 and omega-6 PUFAs in order to maintain low levels of inflammation and to promote cell membrane stability.

## 9. Omega-3 PUFA Supplementation for Prevention of Alzheimer’s Disease and Vascular Cognitive Impairment and Dementia 

The aging brain is more prone to beta-oxidation and inflammatory alterations, that are crucial factors for development of atherosclerotic changes, stroke, VCID, decreased synaptic plasticity and worse memory performance [152]. Omega-3 PUFAs have shown an anti-inflammatory role that may potentially represent a novel treatment for VCID and AD. In a recent study, McGahon et al. showed that dietary supplementation of EPA, DPA and DHA in aged rats decreased inflammation; in fact, the rats showed lower cytokines levels, and had in general positive effects on age-related brain changes [153]. Other studies demonstrated that EPA supplementation was able to counteract the age-related increase in IL-1 levels in the hippocampus of aged rats [154,155]. Similarly, it has been shown that EPA can reduce the increment of hippocampal IL-1 induced by amyloid-β in aged rats [156]. Furthermore, Kelly et al. demonstrated that both EPA and DPA may decrease oxidative stress and may revert age-related spatial learning impairment [157]. Serini et al. compared the anti-inflammatory effect of EPA and DHA in AD patients’ cells and healthy controls cells, documenting a reduced inflammatory cytokines release in cells treated with EPA and DHA [158]. The study described that while DHA has a more powerful effect on decreasing the cytokines released, EPA could more effectively change the pro-inflammatory profile towards one similar to that observed in the healthy controls [158].

Several clinical trials have been conducted with omega-3 PUFAs both in healthy adults and patients with AD or mild cognitive impairment (MCI), using a combination of DHA and EPA. Even if trials conducted on AD patients have yielded negative results, Yurko-Mauro et al. showed significant higher performance in different cognitive domains (paired associated learning and delayed verbal and recognition memory) in healthy adults aged over 55 when treated with 900 mg of DHA for 24 weeks [159]. Lee et al. demonstrated significant improvement in working memory, verbal memory and delayed recall memory in MCI patients aged over 60 when treated with EPA (at a dose of 1.3 g per day) compared to controls [160]. Similarly, Freund-Levi et al. described positive effects in a small group of patients with very mild AD who were treated with EPA and DHA supplements (1.7 g DHA + 0.6 g EPA/daily) [161].

In particular, four RCTs have focused on older adults (Table 2). One trial conducted on Chinese older adults with MCI showed that a combination of 480 mg DHA plus 720 mg EPA/day significantly improved cognitive performances and working memory, compared to placebo [162]. Hooper et al. conducted an RCT on elderly adults showing that 800 mg DHA plus 225 mg EPA/day over a period of 36 months helped to maintain executive function in elderly with low omega-3 index and higher risk of dementia [163]. Boespflug et al. conducted a 6-month trial on older adults with memory impairment and showed that 2.4 g of EPA plus DHA/day significantly improved working memory and neuronal response [164]. Another trial conducted in older adults treated with four 1000 mg omega-3 PUFA supplements daily (containing 1200 mg of EPA plus 800 mg of DHA) showed reduced levels of oxidative stress and thus lower susceptibility to atherosclerosis and VCID [165]. McNamara et al. showed that elderly aged >62 and treated with 24-week fish oil supplementation or blueberry or both had fewer cognitive symptoms, improved memory discrimination and overall improved cognition [166]. Recently, a Canadian study by Power et al. deemed the omega-3 PUFAs and the Mediterranean diet to be key factors in attenuating oxidative damage and inflammation, which are key mechanisms of AD pathogenesis [167]. Taking together, these results from experimental studies and clinical trials suggest as omega-3 PUFAs have a beneficial role on preventing neurodegeneration.

## 10. Novel Personalized Strategies to Prevent Atherosclerosis and Vascular Cognitive Impairment and Dementia: Focus on AA/EPA Ratio and Omega-3 PUFAs

Prevention represents the best way to counteract atherosclerosis and VCID. The main strategy to prevent atherosclerosis and VCID is controlling vascular risk factors by adequate diet and lifestyle, and pharmacologically in case of vascular risk factors and overt disease. The importance of a Mediterranean diet, characterized by the high consumption of fruit, vegetables, legumes, fish, grains and olive oil, along with a low intake of milk, meat, and saturated fatty acids, has since been found to be associated with a significant reduction in risk of cognitive decline [140]. The integration of omega-3 PUFAs on diet has raised a great attention for prevention of atherosclerosis, CVD and VCID, given the causal association between inflammation and such diseases. However, the appropriate omega-3 PUFA dose to reach beneficial effects in terms of primary or secondary prevention of CVD and inflammatory diseases is still unclear. The real benefit of this treatment either with diet or with nutraceutical supplements may depend on the serum and/or tissue levels of omega-3 PUFAs [168]. Thus, various markers of fatty acid intake and status have been proposed. In this regard, it has been shown that omega-6/omega-3 ratio is of limited usefulness for prediction of CV risk, whereas omega-3 index appears to be a more reliable risk factor for CVD. Moreover, the AA/EPA ratio has been shown to be a reliable surrogate marker of omega-6/omega-3 ratio that may serve as a more specific indicator of the magnitude of cellular inflammation and, in turn, CV risk. An AA/EPA ratio between 1.5 and 3 has been suggested as the optimal range in order to reduce cellular inflammation and achieve beneficial effects in different clinical settings [169,170,171,172,173], although larger mechanistic and prospective studies are needed to confirm this hypothesis in the context of CVD and, specifically, VCID. Also, different measurements of AA/EPA ratio have been used across studies, basing on the levels of erythrocyte fatty acids, phospholipid fatty acids, adipose tissue fatty acids, or serum fatty acids. Thus, a standardized method to measure the AA/EPA ratio and relate it to clinical outcomes is also needed. As previously described by Barry Sears in an interesting and recently published editorial [174], the main discrepancies between the studies in terms of clinical benefit of use of omega-3 PUFAs on CV prevention were determined by the variations in the administered dose of omega-3 PUFA supplements. For instance, the dose used in the REDUCE-IT was 4.5 times greater than that used in the VITAL trial. Moreover, it should be worthy to remark that high dose omega-3 PUFAs might increase levels of SPMs and reduce eicosanoid synthesis and pro-inflammatory cytokine expression. Therefore, an accurate dose of omega-3 PUFAs needs to be determined based on several factors. In line with previous and current trials, a dose of approximately 86 mg of EPA and DHA/kg body weight/day has been suggested to have a significant benefit in terms of prevention against CV events [174]. Importantly, a dose of 5 g/day of EPA and DHA has been established to significantly reduce the AA/EPA ratio from 23 to 2.5 in healthy Caucasians, with a corresponding reduction of inflammation [175]. Nonetheless, even if 5 g/day of EPA and DHA represent a proper dose to markedly reduce the AA/EPA ratio, the mean intake of EPA and DHA from supplements and foods in the US is less than 0.3 g/day [176,177]. Therefore, taking also into account the numerous RCTs of omega-3 PUFAs, daily supplementation with 5 g of EPA and DHA needs to be considered in an experimental setting. In general, a daily dose of EPA and DHA of 2.5 g seems to be sufficient to lower the AA/EPA ratio within the desired range for excellent wellness for healthy individuals [173]. The ability of AA/EPA ratio to give a parameter of omega-3 PUFA treatment in terms of anti-inflammatory effects may allow for development of a personalized therapy for inflammatory-based diseases, including atherosclerosis and VCID. Omega-3 PUFAs may induce an early prevention by acting at different levels of VCID pathogenesis, including reducing vascular inflammation, and decreasing noxious molecular pathways activation leading to accumulation of misfolded proteins in the neurons. Therefore, therapy with omega-3 PUFAs, especially in subjects with a high risk of VCID—such as patients with diabetes, hypertension and dyslipidemia—may significantly benefit from this treatment.

Moreover, other inflammatory parameters have been associated with risk for vascular diseases, such as high-sensitivity C-reactive protein (hs-CRP), IL-6 dominant inflammation, and lipoprotein-associated phospholipase A2 (Lp-PLA2) in large cohorts, including the Northern Manhattan Study (NOMAS) [178,179]. It is important to mention that so far, studies that investigated the activity of darapladib against PLA2 activity, did not show direct reduction of CV events when added to optimal medical treatment [180,181,182]. However, several studies have shown how omega-3 PUFA supplementation decreases the risk of AD and cognitive impairment. This may be due to the different activity of PLA2 in the brain or to the pleiotropic effect that omega-3 PUFAs exert on the brain tissue. 

It is also worth noting that majority of clinical trials have been conducted on Caucasians. Hence, an optimal AA/EPA ratio level and adjusted omega-3 PUFA treatment should be investigated in other race-ethnic groups, given the high risk to develop CVD [183]. Finally, future studies will be necessary in order to compare the sensitivity and specificity of the AA/EPA ratio to those of other inflammatory markers (e.g., CRP), and also to address whether the AA/EPA ratio may be useful in vascular risk prediction, along with hs-CRP, IL-6 and Lp-PLA2. 

## 11. Conclusions

Although the role of omega-6 PUFAs in triggering systemic inflammation remains controversial, growing evidence highlights the importance of increasing the absolute intake of omega-3 PUFAs in order to reduce CV risk. Indeed, the beneficial CV effects of omega-3 PUFAs may rely on their anti-inflammatory and anti-atherosclerotic properties. 

Vascular contribution to VCID is frequently underestimated, both in terms of disease burden and potential for understanding and preventing dementia. Converging data from clinical, neuropathological and experimental studies have begun to unravel the association between white matter hyperintensities (WMH) and VCID, and have uncovered substantial advances in the molecular understanding and clinical management of WMH and VCID. A deeper molecular understanding has also opened the possibility for novel therapeutic agents as the omega-3 PUFAs [31,32]. Currently, the management of WMH of vascular origin is limited to lifestyle modifications and risk factor control. Given the associations between WMH and vascular risk factors, it is pivotal to target vascular health throughout the life course as a prevention strategy. Effective regulations on the content of foods and diet, that include omega-3 PUFAs, may offer an important therapeutic option [31,32].

With regard to the biomarkers of fatty acid intake and status, omega-3 index and AA/EPA ratio appear to be potentially useful for measuring the burden of systemic inflammatory diseases and predicting and/or modifying CV and VCID risk. Notably, titrating the dose of supplementary omega-3 PUFAs based on the values of these biomarkers might represent an additional strategy to prevent CVD and VCID. However, mechanistic studies and large prospective trials are warranted in order to confirm the clinical usefulness of omega-3 index and AA/EPA ratio as risk factors for CV and VCID, due to the current lack of robust evidence-based literature. In conclusion, experimental and clinical studies are awaited to establish the efficacy of omega-3 PUFA supplementation at a proper dose as a successful prevention strategy against atherosclerosis, CVD and VCID. 

## Figures and Tables

**Table 1:** Summary of the main randomized controlled trials assessing the efficacy of omega-3 PUFAs in primary and secondary prevention of cardiovascular disease and vascular cognitive impairment and dementia. Abbreviations: ALA, alpha-linolenic acid; CAD, coronary artery disease; CI, confidence interval; CV, cardiovascular; CVD, cardiovascular disease; EPA, eicosapentaenoic acid; DHA, docosahexaenoic acid; hsCRP, high-sensitivity C-reactive protein; MI, myocardial infarction; PUFA, polyunsaturated fatty acids.

	Study Population	Study Duration	Omega-3 PUFA dose	Clinical Findings
JELIS [103]	18,645 patients with hypercholesterolemia and with/without history of CAD	Mean follow-up: 4.6 years	EPA 1800 mg/day + statin vs. statin alone	Reduction in major coronary events in the EPA group compared to control in the total study population (hazard ratio, 0.81; 95% CI, 0.69 to 0.95; *p* = 0.011) Reduction in major coronary events in the EPA group compared to control group among patients with history of CAD (hazard ratio, 0.81; 95% CI, 0.66 to 1.00; *p* = 0.048)
Alpha Omega Trial [106]	4837 subjects with history of MI, who were receiving state-of-the-art antithrombotic, antihypertensive, and lipid-lowering therapy	Median follow-up: 40.8 months	Margarine enriched with EPA and DHA (400 mg of EPA and DHA/day) or ALA (2 g of ALA/day)	Rate of major CV events: hazard ratio with EPA-DHA-enriched margarine, 1.01; 95% CI, 0.87 to 1.17; *p* = 0.93; hazard ratio with ALA-enriched margarine, 0.91; 95% CI, 0.78 to 1.05; *p* = 0.20
SU.FOL.OM3 [107]	2501 patients with a history of unstable angina, MI, or ischemic stroke	Median follow-up: 4.7 years	600 mg/day of EPA and DHA in a ratio of 2:1 vs. placebo	No significant difference in major CV events between omega-3 group and placebo group (81 vs. 76 patients, hazard ratio 1.08; 95% CI 0.79 to 1.47, *p* = 0.64)
ORIGIN [108]	12,536 participants with diabetes, impaired glucose tolerance or impaired fasting glucose, who were at increased CV risk, defined as history of MI, angina with documented ischemia, stroke, or revascularization	Median follow-up: 6.2 years	465 mg of EPA/day plus 375 mg of DHA/day vs. placebo	No significant difference in death from CV causes between omega-3 group and placebo group (9.1% vs. 9.3%; hazard ratio, 0.98; 95% CI, 0.87 to 1.10; *p* = 0.72) No significant difference between omega-3 group and placebo group in the rate of major CV events (16.5% *vs.* 16.3%; hazard ratio, 1.01; 95% CI, 0.93 to 1.10; *p* = 0.81), death from arrhythmia (4.6% *vs.* 4.1%; hazard ratio, 1.10; 95% CI, 0.93 to 1.30; *p* = 0.26), or death from any cause (15.1% vs. 15.4%; hazard ratio, 0.98; 95% CI, 0.89 to 1.07; *p* = 0.63)
Risk and Prevention Study [109]	12,513 patients with multiple CV risk factors or clinical evidence of atherosclerotic vascular disease (defined as a history of transient ischemic attack or ischemic stroke, angina pectoris, peripheral artery disease, or previous arterial revascularization procedure) without history of previous MI	Median follow-up: 5 years	1-g daily capsule containing not less than 85% of EPA and DHA content and in a ratio ranging between 0.9:1 and 1.5:1 vs. placebo	No significant difference in time to death from CV causes or first hospitalization for CV causes between omega-3 group and placebo group (11.7% vs. 11.9%; adjusted hazard ratio with n-3 fatty acids, 0.97; 95% CI, 0.88 to 1.08; *p* = 0.58)
ASCEND [110]	15,480 participants with diabetes and no evidence of CVD	Mean follow-up: 7.4 years	460 mg of EPA/day plus 380 mg of DHA/day vs. placebo	No significant difference in first serious vascular event between omega-3 group and placebo group (8.9% vs. 9.2%; rate ratio, 0.97; 95% CI, 0.87 to 1.08; *p* = 0.55) No significant difference in the secondary composite outcome of any serious vascular event or any revascularization procedure between omega-3 group and placebo group (11.4% vs. 11.5%; rate ratio, 1.00; 95% CI, 0.91 to 1.09)
VITAL [111]	25,871 participants without history of CVD	Median follow-up: 5.3 years	460 mg of EPA/day plus 380 mg of DHA/day vs. placebo	No significant difference in the incidence of major CV events between omega-3 group and placebo group (hazard ratio, 0.92; 95% CI, 0.80 to 1.06; *p* = 0.24) Omega-3 PUFA supplementation associated with a significant reduction in risk of total MI compared to placebo (hazard ratio, 0.72; 95% CI, 0.59 to 0.90)
REDUCE-IT [112]	8179 patients with hypertriglyceridemia and established CVD or diabetes and other risk factors (70.7% for secondary prevention of CV events)	Median follow-up: 4.9 years	4 g/day of icosapent ethyl, a highly purified EPA ethyl ester vs. placebo	Significant reduction in the rates of the primary endpoint (a composite of CV death, non-fatal MI, non-fatal stroke, coronary revascularization, or unstable angina) in the icosapent ethyl group compared to placebo group (hazard ratio, 0.75; 95% CI, 0.68 to 0.83; *p* < 0.001) Significant reduction in the rates of the key secondary endpoint (a composite of CV death, non-fatal MI, or non-fatal stroke) in the icosapent ethyl group compared to placebo group (hazard ratio, 0.74; 95% CI, 0.65 to 0.83; *p* < 0.001) Significant reduction in circulating levels of hsCRP in the icosapent ethyl group compared to placebo group (median observed values at the last follow-up visit: 1.8 mg/L vs. 2.8 mg/L, respectively; *p* < 0.001)

**Table 2:** Summary of the main randomized controlled trials assessing the efficacy of omega-3 PUFAs in prevention of vascular cognitive impairment and dementia. Abbreviations: EPA, eicosapentaenoic acid; DHA, docosahexaenoic acid; MCI, mild cognitive impairment; PUFA, polyunsaturated fatty acids.

	Study Population	Study Duration	Omega-3 PUFA dose	Clinical Findings
Duffy et al. [165]	51 heathy older adults (mean age = 71 years)	12 weeks	Four 1000 mg omega-3 supplements (containing EPA 1200 mg + DHA 800 mg) daily	The participants treated with the omega-3 supplements had lower oxidative stress (measured as higher glutathione-to-creatine ratio in the thalamus) compared to the placebo group (*p* = 0.049)
Boespflug et al. [164]	140 healthy adults aged 62–80 years with subjective memory complaints, but not meeting criteria for MCI or dementia	24 weeks	EPA + DHA at a dose of 2.4 g/day	Dietary fish oil supplementation ameliorated working memory performance, and enhanced neuronal response to working memory challenge (defined as increased blood oxygen level dependent signal in the posterior cingulate cortex during greater working memory load)
Hooper et al. [163]	183 adults aged 70 years or older with subjective memory complaints but clinically dementia-free	36 months	Two capsules of omega-3 supplement providing a total 800 mg DHA + 225 mg EPA daily	Omega-3 PUFAs showed benefits in maintenance of executive functions in older adults at risk of dementia due to low omega-3 index
Bo et al. [162]	86 adults with mean age 71 years affected by MCI	6 months	Omega-3 PUFA supplement capsules of 480 mg DHA + 720 mg EPA/daily	Omega-3 PUFA supplementation was associated with improved total Basic Cognitive Aptitude Test scores, space imagery efficiency, processing speed, and working memory (*p* < 0.01)
